# Parsing altered gray matter morphology of depression using a framework integrating the normative model and non-negative matrix factorization

**DOI:** 10.1038/s41467-023-39861-z

**Published:** 2023-07-08

**Authors:** Shaoqiang Han, Qian Cui, Ruiping Zheng, Shuying Li, Bingqian Zhou, Keke Fang, Wei Sheng, Baohong Wen, Liang Liu, Yarui Wei, Huafu Chen, Yuan Chen, Jingliang Cheng, Yong Zhang

**Affiliations:** 1 Department of Magnetic Resonance Imaging, The First Affiliated Hospital of Zhengzhou University, Henan Province, China; 2 Key Laboratory for Functional Magnetic Resonance Imaging and Molecular Imaging of Henan Province, Henan Province, China; 3 Engineering Technology Research Center for Detection and Application of Brain Function of Henan Province, Henan Province, China; 4 Engineering Research Center of Medical Imaging Intelligent Diagnosis and Treatment of Henan Province, Henan Province, China; 5 Key Laboratory of Magnetic Resonance and Brain Function of Henan Province, Henan Province, China; 6 Key Laboratory of Brain Function and Cognitive Magnetic Resonance Imaging of Zhengzhou, Henan Province, China; 7 Key Laboratory of Imaging Intelligence Research medicine of Henan Province, Henan Province, China; 8 Henan Engineering Research Center of Brain Function Development and Application, Henan Province, China; 9 School of Public Affairs and Administration, University of Electronic Science and Technology of China, Chengdu, China; 10 Department of Psychiatry, The First Affiliated Hospital of Zhengzhou University, Henan Province, China; 11 Department of Pharmacy, Affiliated Cancer Hospital of Zhengzhou University, Henan Cancer Hospital, Henan Province, China; 12 The Clinical Hospital of Chengdu Brain Science Institute, School of Life Science and Technology, University of Electronic Science and Technology of China, Chengdu, China

**Keywords:** Depression, Neuroscience, Depression

## Abstract

The high inter-individual heterogeneity in individuals with depression limits neuroimaging studies with case-control approaches to identify promising biomarkers for individualized clinical decision-making. We put forward a framework integrating the normative model and non-negative matrix factorization (NMF) to quantitatively assess altered gray matter morphology in depression from a dimensional perspective. The proposed framework parses altered gray matter morphology into overlapping latent disease factors, and assigns patients distinct factor compositions, thus preserving inter-individual variability. We identified four robust disease factors with distinct clinical symptoms and cognitive processes in depression. In addition, we showed the quantitative relationship between the group-level gray matter morphological differences and disease factors. Furthermore, this framework significantly predicted factor compositions of patients in an independent dataset. The framework provides an approach to resolve neuroanatomical heterogeneity in depression.

## Abstract (web summary)

The neuroanatomical heterogeneity of depression is not well understood. Here, the authors identify four latent factors which characterise different patterns of gray matter morphology and are related to clinical symptoms.

## Introduction

It is well accepted that depression is a highly heterogeneous syndrome. Patients with depression show pronounced inter-individual variations in symptom manifestation, clinical trajectories, etiologies, and treatment responses^1–4^. Two patients diagnosed with depression may experience very different (or even opposite) symptom profiles^5^, have different monoamine levels^6^ and respond differently to treatment^7^. Interindividual heterogeneity hampers neuroimaging studies with case-control approaches to identify promising biomarkers for individualized clinical decision-making. This is because traditional neuroimaging studies aim to detect group-level effects but ignoring inter-individual heterogeneity^8^. Neuroimaging studies establish that group-level differences essentially representing an “average patient” are on behalf of a fraction of patients^8^ and miss important inter-individual heterogeneity^9^. In this context, although studies using structural magnetic resonance imaging (MRI) consistently recognize structural brain abnormalities in distributed brain regions, their findings are not uniform because they adopt almost exclusively case-control approaches in depression^10^. Parsing neuroanatomical heterogeneity not only provides insights into the etiology, but also facilitates individualized clinical decision-making in depression.

An increasing number of researchers have realized neuroanatomical heterogeneity and have proposed a series of approaches to resolve it. These approaches can be roughly summarized into two categories: identifying more homogeneous subtypes and obtaining subject-level differences. The former aims to uncover potential subtypes. Clinically, patients with depression are usually divided into non-overlapping/categorical subtypes according to symptomatology, course trajectories, and treatment responses^11^. Alternatively, with advances in machine learning and the availability of open datasets, researchers have revealed potential subtypes using data-driven methods from objective data, such as biological and neuroimaging data^12^. Although these approaches show promise in practical clinical applications to a certain extent, such as informing treatment responses^13^ and treatment selection^14^, they also have limitations. Similar symptoms can be introduced by distinct mechanisms, restricting the application of them in distinguishing etiologically distinct patients^15^. Importantly, some patients may not clearly belong to any subtype, and subtypes according to symptoms is unstable over time^16^. Data-driven methods also encounter a number of challenges, e.g., choosing clustering algorithms and determining the optimal number of subgroups^17,18^. Findings derived from subtypes still cannot be applied to an individual patient. The latter obtains neuroimaging phenotype at subject level. Recently, a normative model is proposed to evaluate subject-level imaging differences by measuring the deviation from the normal distribution^18^. With the normative model, neuroimaging studies succeed in inferring subject-level structural brain alterations in mental disorders^8,19,20^. Nonetheless, the high- dimension results derived from the normative model is difficult to deal with^8,19^. On the other hand, relevant neuroimaging studies dot not reconcile subject- to group-level findings, limiting interpretability of subject-level results.

**Fig. 1:**
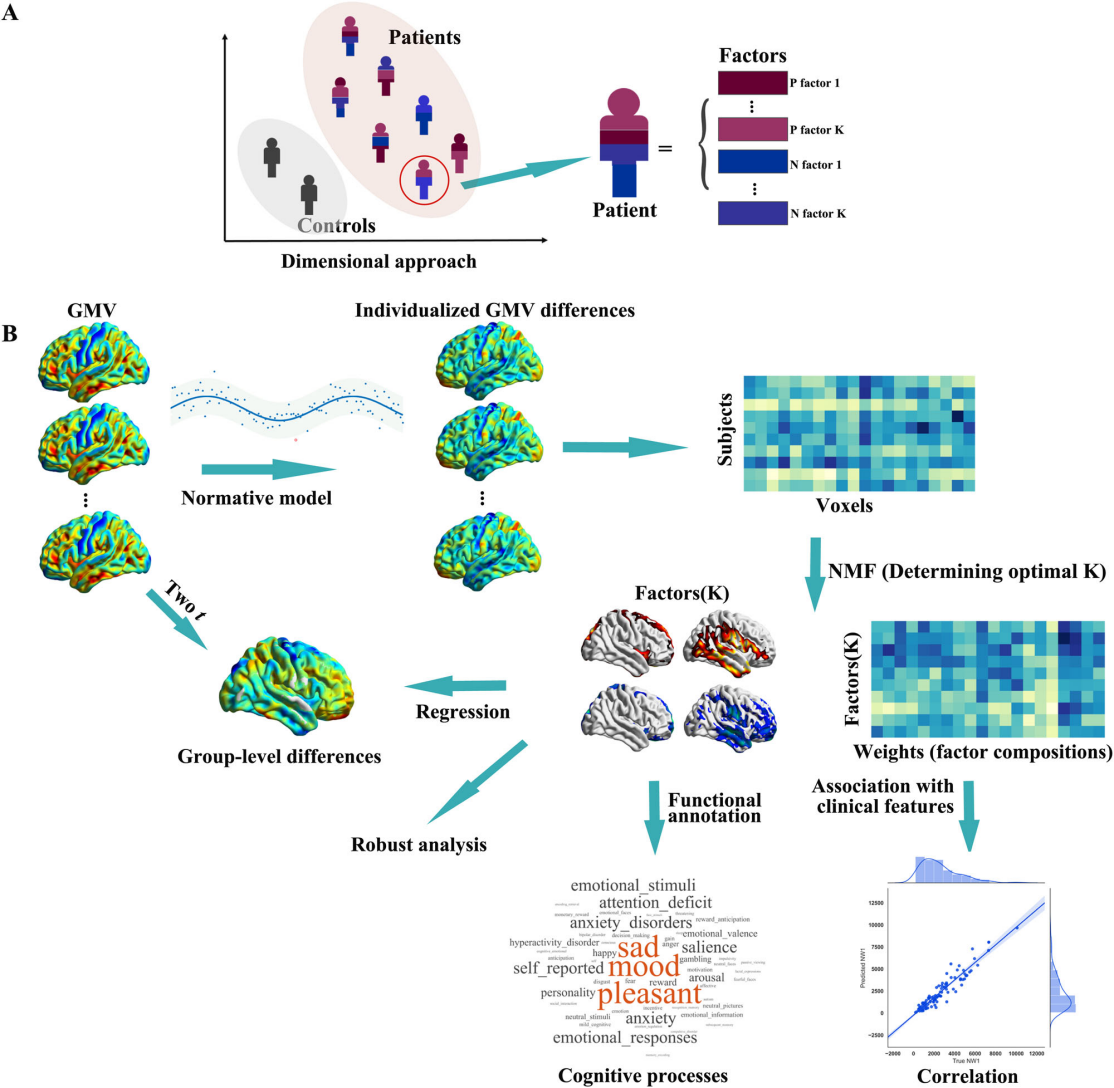
Study overview. **A** Illustration of categorical approach and dimensional approach. **B** The flow chart of this study. The subject-level gray matter volume (GMV) differences are obtained with the normative model and parsed into latent disease factors (and factor composition) with the optimal number (K) using NMF. Then, we associate factor composition (weights) with clinical symptoms and perform functional annotation on the identified disease factors. Robust analysis is conducted. Finally, investigating the association between subject- and group-level differences. Two *t*, two sample *t* test; NMF non-negative matrix factorization.

In this study, we propose a comprehensible framework to quantitatively estimate structural brain abnormalities from a dimensional perspective in depression. In this framework, subject-level gray matter morphological differences measured with voxel-based morphometry analysis were derived using the normative model^8,21^. Then non-negative matrix factorization (NMF), a widely used dimensionality reduction algorithm in neuroimaging studies^22,23^, was conducted to parse subject-level differences into latent disease factors. In this way, subject-level morphological differences are expressed as a linear and unique combination of disease factors (Fig. 1A). We then performed functional annotation on identified disease factors to associate them with functional/cognitive terms using probabilistic (activation) mappings provided by Neurosynth (https://neurosynth.org/)^24^. In this study, we had two main hypotheses: (1) Individual differences in clinical presentation could be reflected in the factor composition; (2) The disease factors could quantitatively derive the group-level morphological results. What’s more, we also investigated the reproducibility of the framework in another independent dataset.

## Results

### Overall approach

This study included three independent datasets: a discovery dataset and two validation datasets. The discovery dataset recruited 105 patients with depression. The validation dataset 1 included 76 patients with depression and 68 HCs and the validation dataset 2 included 492 HCs. All reported results were based on the discovery dataset and validated using the validation datasets.

The workflow of this study involved five steps (Fig. 1B). (1) Performing the normative model to derive individualized gray matter volume (GMV) differences. (2) Performing NMF to parse subject-level GMV differences into disease factors and factor composition. In this step a strategy was also proposed to automatically identify the optimal number of disease factors (K). (3) Investigating the relationship between factor composition and symptoms, and performing functional annotation on disease factors. (4) Reproducibility analysis. (5) Association between subject- and group-level differences were investigated Table 1.

**Table 1:** Clinicodemographic characteristics

	Discovery dataset			Validation dataset 1			Validation dataset 2
	Depression (*N* = 105)	HCs (*N* = 130)	*p*	Depression (*N* = 76)	HCs (*N* = 68)	*p*	HCs
Male, No. (%)	50.48%	45.38%	0.963^a^	39.47%	51.47%	0.843^a^	38.01%
Age, mean (SD), [range], y	20.30 (5.04) [11–37]	21.05 (5.33) [12–36]	0.256^b^	24.76 (6.58) [14–37]	25.06 (5.12) [18–37]	0.766^b^	45.10 (17.30) [19–80]
Educational level, mean (SD), y	12.81 (5.13)	13.56 (4.49)	0.230^b^	13.51 (2.76)	16.38 (2.19)	2.106e^−10 b^	
HAMD, mean (SD)	22.26 (6.19)			27.60 (7.87)			
Age of onset, mean (SD), y	18.83 (4.94)			28.69 (11.55)			
Anxiety/somatization, mean (SD)	5.77 (2.56)						
Weight, mean (SD)	0.15 (0.51)						
Cognitive impairment, mean (SD)	2.92 (2.46)						
Psychomotor slowing, mean (SD)	8.55 (2.00)						
Sleep disturbance, mean (SD)	4.21 (2.25)						
IQR, mean (SD)	1.98 (0.14)	2.09 (0.29)	<0.001^b^	1.99 (0.10)	1.98 (0.11)	0.759^b^	

*HC* healthy control, *HAMD* Hamilton rating scale for depression, ^a^ χ2 test, ^b^ two-sided two-sample *t* test, *IQR* Image Quality Rating.

### Four disease factors are identified

**Fig. 2:**
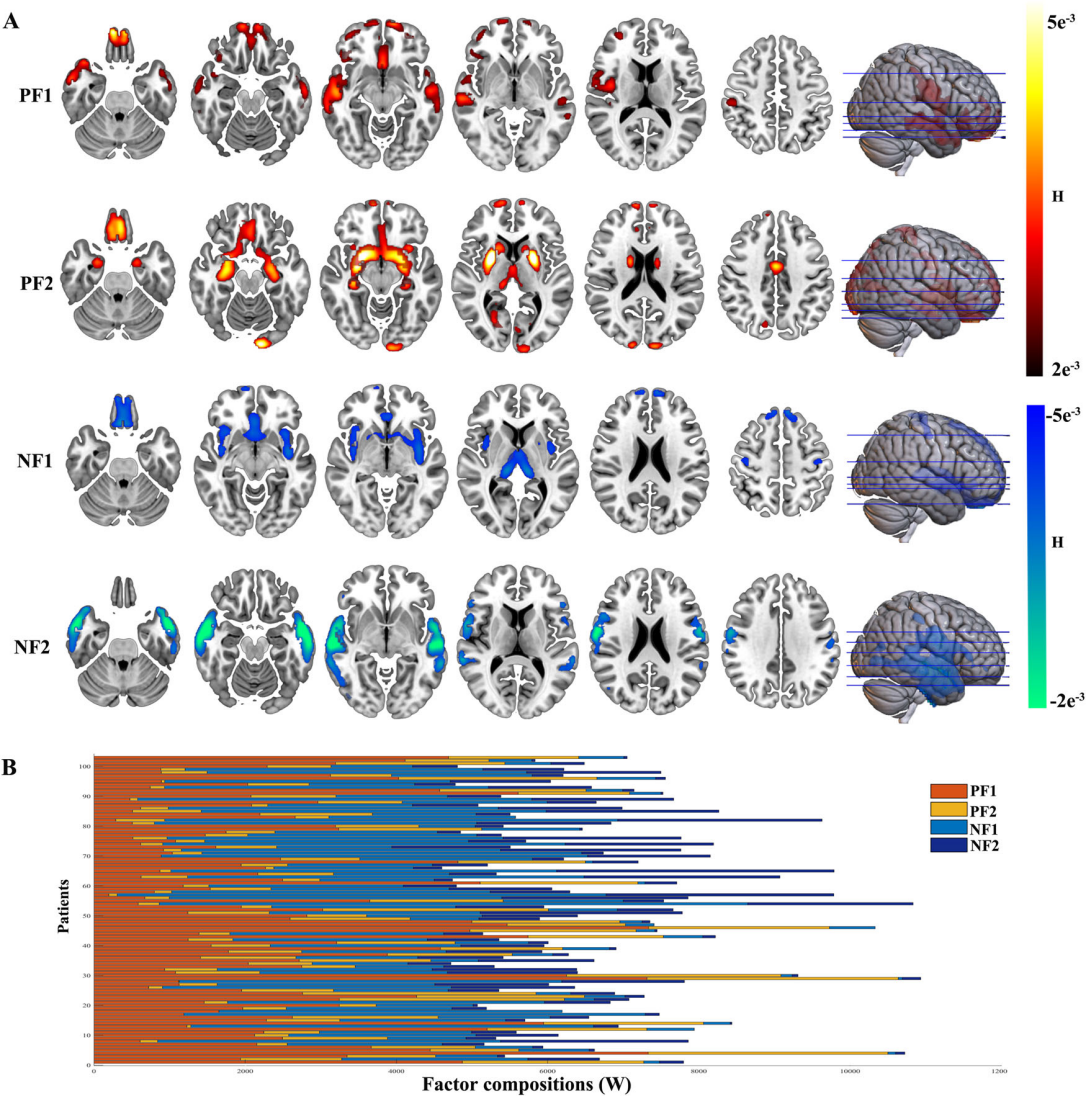
The identified four latent disease factors (H) and the factor composition (W). **A** The distribution of the identified four latent disease factors (K). The top 5% voxels according to H values are shown. **B** The factor composition (W values) for each patient. PF1 positive factor 1, PF2 positive factor 2, NF1 negative factor 1, NF2 negative factor 2. Source data are provided as a Source Data file.

We identified disease factors of subject-level differences using NMF. To choose the appropriate number of disease factors, an index named stability value was proposed with the assumption results of the optimal number of disease factors were the most stable (see method). A larger stability value indicates a more stable result. The stability values of the increased and decreased parts of the two datasets are presented in Fig. S1. As shown, the stability value was the largest when the number of disease factors (K) = 2 (for increased and decreased differences), suggesting there were two positive and two negative disease factors. The permutation test results (see method) suggested that NMF would yield more stable results and explain greater variance than chance (Supplementary results). These four disease factors are shown in Fig. 2A. Only the top 5% of voxels according to disease factors (H) for visualization are shown. The factor compositions (W) of patients are shown in Fig. 2B. Positive disease factor 1 comprised the orbital frontal cortex, ventromedial prefrontal cortex/anterior cingulate cortex, temporal pole, and middle temporal gyrus. Positive disease factor 2 comprised the striatum, ventromedial prefrontal cortex (vmPFC)/anterior cingulate cortex (ACC), parahippocampal gyrus, thalamus, and amygdala. Negative disease factor 1 comprised the vmPFC/ACC, superior frontal gyrus, bilateral anterior insula, thalamus, striatum, hippocampus, and precentral gyrus. Negative disease factor 2 comprised the bilateral temporal gyrus, inferior frontal gyrus, and postcentral gyrus. The identified four disease factors in the validation dataset 1 and their spatial correlations with these in the discovery dataset were presented in Fig. S2.

Patients with depression showed large variation in factor composition (Fig. 2B). We embedded factor composition (four weight values) into two-dimensional space with a dimensionality reduction technique (*t*-distributed stochastic neighbor embedding method)^25^. These results intuitively indicated that patients with depression exhibited high heterogeneity in factor composition.

### Functional annotation results

Functional annotation identified a series of cognitive/functional terms for each disease factor from the 217 terms with clear biological significance. Overall, positive factor 1 was mainly related to autobiographical memory, positive factor 2 was to mood and attentional deficits, negative factor 1 was to pressure, and conscious and negative factor 2 was to language comprehension and communication. Significantly related terms (permutation *p* < 0.05, FDR corrected) were included in the word cloud (Fig. S3 and Table S1), where the size of the word was proportional to 1/permutation *p*. The functional annotation results of the validation dataset 1 are presented in Table S2.

### Association between factor composition and clinical characteristics

**Fig. 3:**
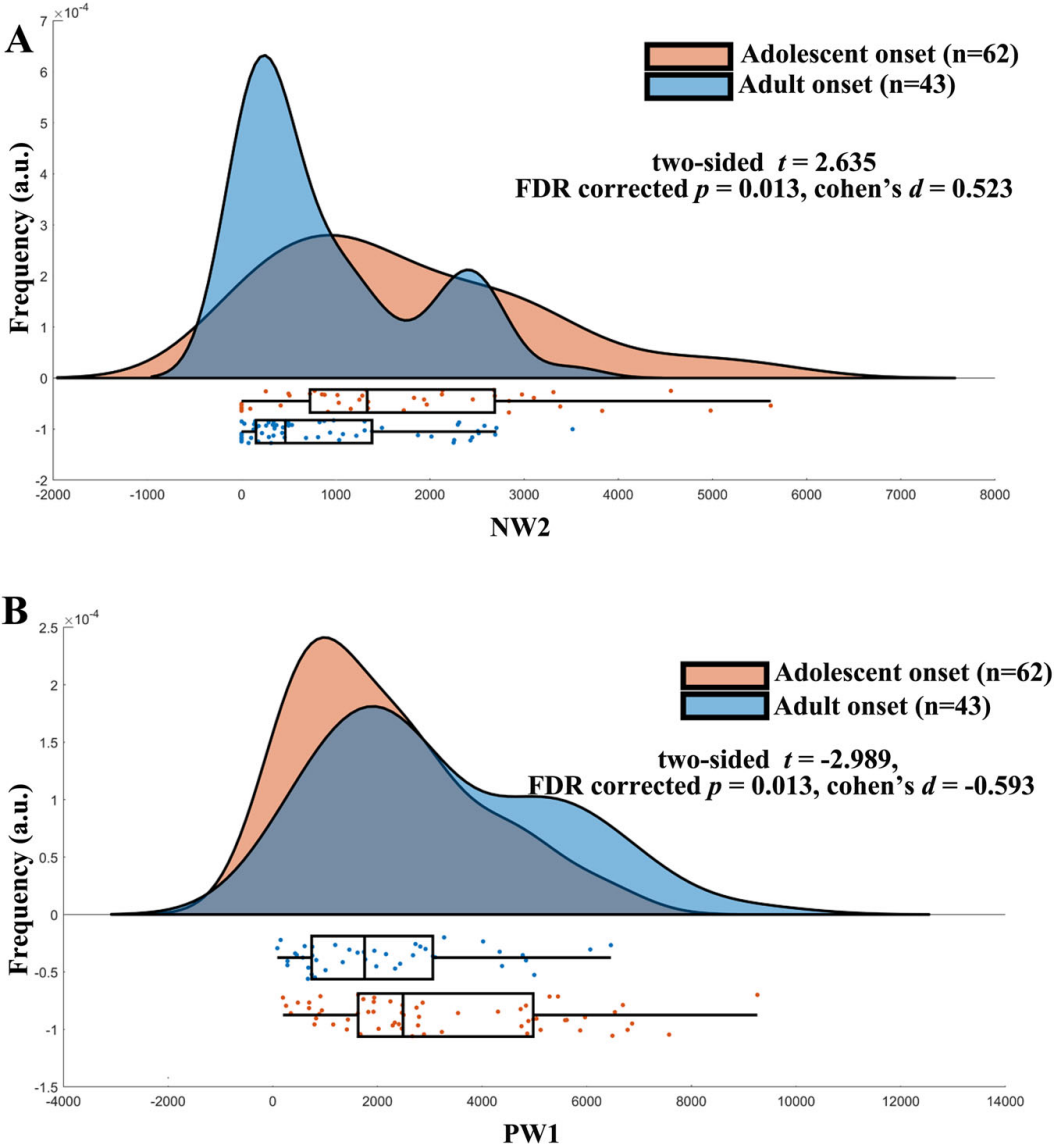
Differences of factor composition between adult-onset and adolescent-onset patients. Adult onset patients have significantly larger NW2 (**A**) and smaller PW1 (**B**) than adolescent-onset patients. The y-axes represent the frequency, and the unit is arbitrary (a.u.). Box plots represent median value, first and third quartiles; whiskers represent the empirical 95% confidence interval. PW1 weight of positive factor 1, NW2 weight of negative factor 2. Source data are provided as a Source Data file.

We did not observe any correlations between factor composition and clinical symptoms (FDR corrected *p* values < 0.05). There was also no significant correlation between educational level and the weight of disease factors, further excluding the effect of educational level on the results. Further, the weights of the disease factors demonstrated no difference between male and female patients (Weights of positive factor 1 (PW1): *t* = 0.778, *p* = 0.438; Weights of positive factor 2 (PW2): *t* = 1.549, *p* = 0.124; Weights of negative factor 1 (NW1): *t* = −0.137, *p* = 0.892; Weights of negative factor 2 (NW2): *t* = 1.567, *p* = 0.120). However, there were significant differences in NW2 (adult onset vs. adolescent onset: *t* = 2.635, FDR corrected *p* = 0.013, Cohen’s *d* = 0.523, Fig. 3A) and PW1 (adult onset vs. adolescent onset: *t* = −2.989, FDR corrected *p* = 0.013, Cohen’s *d* = −0.593, Fig. 3B) between adult-onset patients (age >18 years) and adolescent-onset patients (age ≤18 years).

### Robust analysis results

**Fig. 4:**
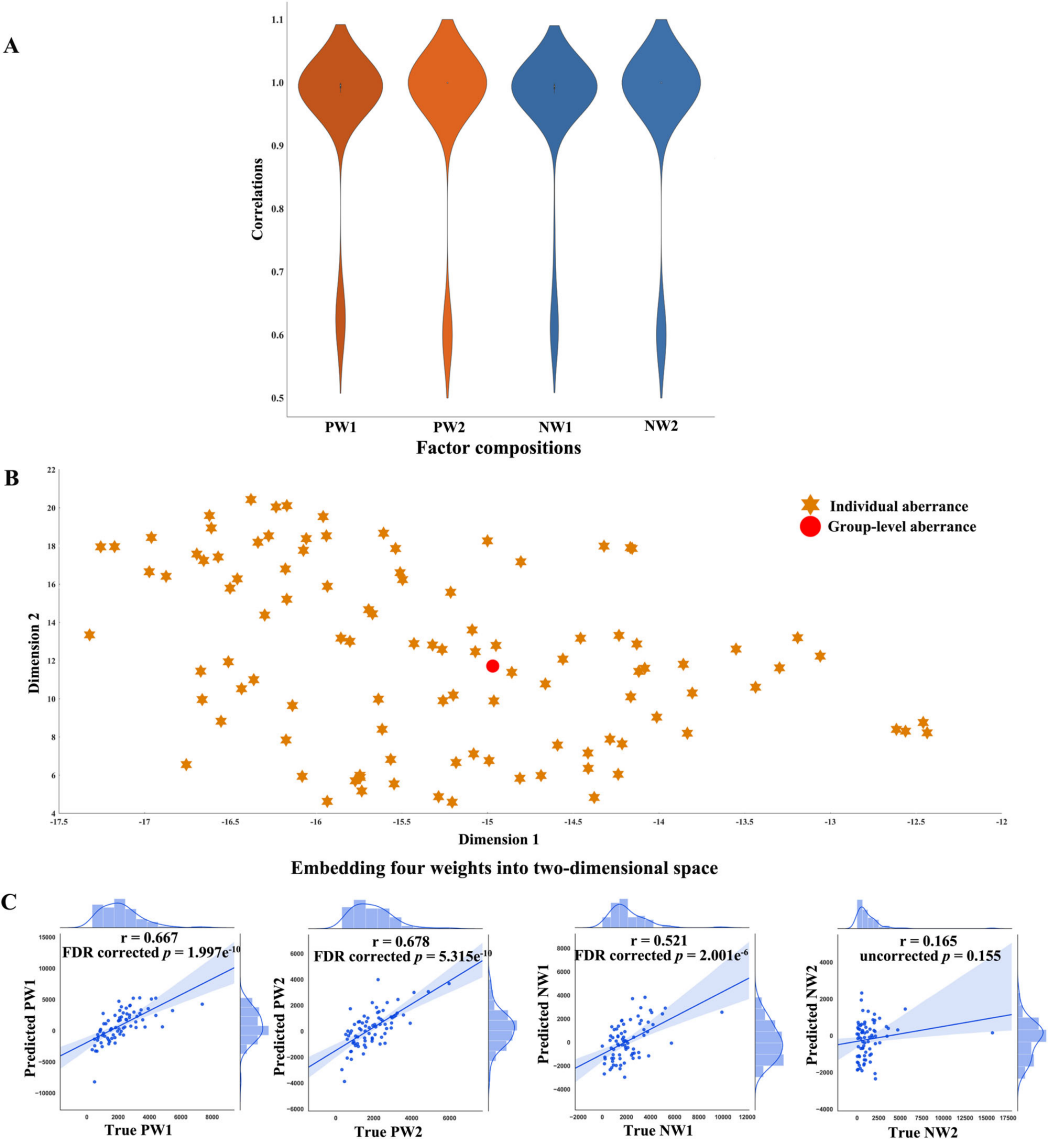
Reproducibility analysis results and association between subject-level and group-level differences. **A** Distribution of the correlation coefficients between the four disease factors identified using the overall patients and that using a patient subset (90%) (*N* = 100). **B** Factor composition of all patients is embedded into 4 into two-dimensional space. The orange hexagram represents subject-level differences of each patient, and the red circle represents group-level differences (“average” patient). **C** The correlations between the predicted weights and the true weights in the validation dataset 1. Shadow represent the empirical 95% confidence interval. PW1 weight of positive factor 1, PW2 weight of positive factor 2, NW1 weight of negative factor 1, NW2 weight of negative factor 2. Source data are provided as a Source Data file.

We also investigated the robustness of the identified disease factors by running NMF 100 times on a randomly selected population of 90% of the patients. Spearman’s correlation coefficients between the factors in the overall patients and in a patient subgroup are shown in Fig. 4A. As we found that patients demonstrated significantly lower IQR than HCs, to exclude the potential effect of IQR on our results, we also calculated Spearman’s correlation coefficients between IQR and factor composition. As a result, no significant correlation was observed (uncorrected *p* values > 0.05).

### Framework prediction of factor composition of unseen patients

The framework could significantly predict the factor composition of patients in the independent validation dataset 1 (r = 0.667, *p* < 0.001 for PW1; r = 0.678, *p* < 0.001 for PW2; r = 0.521, *p* < 0.001 for NW1; r = 0.165, *p* = 0.154 for NW2) (Fig. 4C).

### The quantitative relationship between group-level results and disease factors

The group-level gray matter morphological differences of the discovery dataset are shown in Fig. S4. Patients with depression exhibited decreased GMV in certain brain regions, including the thalamus, hippocampus, parahippocampus, and precentral gyrus (*p* < 0.05, FDR corrected). These was a significantly association between the group-level results and the identified 4 disease factors using a linear regression model (R^2^ = 0.738, F = 2.355 × 10^5^, *p* < 0.001):

1 $${{{{{\rm{Group}}}}}}-{{{{{\rm{level\; differences}}}}}}\,=\, 1.488\times {10}^{3}*{{{{{{\rm{factor}}}}}}}_{{{{{{\rm{p}}}}}}1}+0.637\times {10}^{3}*{{{{{{\rm{factor}}}}}}}_{{{{{{\rm{p}}}}}}2}\\ {-}1.412\times {10}^{3}*{{{{{{\rm{factor}}}}}}}_{{{{{{\rm{nl}}}}}}}{-}0.663\times {10}^{3}*{{{{{{\rm{factor}}}}}}}_{{{{{{\rm{n}}}}}}2}$$

In addition, to intuitively show the relationship between the subject- and group-level results. Group-level results were assumed to obtained from an ‘average’ patient and the coefficients (obtained before) were treated as its factor composition. Factor composition of the “average” patient along with that of each patient were embedded into a two-dimensional space (Fig. 4B). This relationship was validated in the validation dataset 1 (supplementary results).

## Discussion

In the current study, we put forward a comprehensible framework to quantitatively assess altered gray matter morphological of patients with depression. This framework parses subject-level gray matter morphological differences into latent disease factors and retains inter-individual variability. Factor composition significantly differs between adult- and adolescent-onset patients. In addition, identified disease factors can significantly derive group-level gray matter morphological differences. Moreover, the framework can predict the factor composition of patients in another independent dataset.

The normative model can map individualized brain morphological differences but yield high-dimension results at the same time which is hard to handle with^8,19^. In this study, we go further to parse subject-level morphological differences into disease factors using NMF that is found to be able to uncover disease factors in autism spectrum disorder^23,26^. In the work of Shan et al., the authors also decompose gray matter matrix into six disease factors using NMF and obtain individualized deviations of weight using normative model for each patient with autism spectrum disorder^23^. However, there are three main differences between their work and the current study. First, the assumption is different. In the framework used in autism, the authors do not directly obtain disease-specific factors, but the different compositions of factors shared by patients and normal population in autism. Second, the number of disease factors is automatically determined in our proposed framework. Finally, quantitative relation between subject- and group-level morphological results is also identified, reconciling the group- and subject-level results. We tackle inter-individual gray matter morphological heterogeneity in depression from a dimensional prospective, in accordance with dimension model of psychiatric disorders^27^.

Previous similar studies^23,28,29^ fails to obtain disease-related differential patterns at subject level or validate the reproducibility of their findings. Our proposed framework identifies four robust disease factors derived from individualized morphological differences in patients with depression. The identified disease factors demonstrate distinct spatial distributions and cognitive processes. Although the identified factors are significantly associated with cognitive processes, we don’t find any significant correlations between the weights of disease factors and symptoms. The possible explanations are numerous: First, ‘mood’ in the Neurosynth is a very broad term including neutral, sad, and happy mood states derived from both patients with brain disorders (mainly mental disorders) and healthy population. Not all of them are related to depression. Second, the structural/functional neural correlations of the same clinical symptom vary between patients with depression and healthy population or patients with other mental disorders, such as anhedonia^30–32^. Finally, we don’t record enough clinical measures. Considering the association between age of onset and disease factors, clinical measures, such as antidepressant treatment response^33^, executive dysfunction^34^, prevalence of stressful life events preceding onset and levels of neuroticism^35^ may also be associated with disease factors.

The patients with depression exhibit tremendous inter-individual heterogeneity in factor composition. Examination of factor composition show that patients with depression are uniformly distributed in the embedded space. Although medical status and comorbidity are well controlled in this study, there is still notable heterogeneity in factor composition among patients with depression, in accordance with the notion that depression is a highly heterogeneous disorder^36,37^. In addition, factor composition demonstrates significant differences between adult- and adolescent-onset patients. The age of onset has been consistently recognized as one of the sources of clinical heterogeneity and import potential confounders, leading to conflicting findings of neuroimaging studies in depression^10,33,38–40^. For example, unlike adult-onset depression characterized by decreased volume in widespread brain regions^41^, adolescent-onset depression demonstrates increased volume in distributed brain regions such as the hippocampus, amygdala, and anterior cingulate cortex, possibly reflecting an delayed dendritic pruning^42,43^. Collectively, these results suggested that our framework uncovered potential disease factors that captured the sources of clinical heterogeneity in depression. Meanwhile, the personalized and multifarious factor composition provided a possible interpretation of the conflicting findings of case-control neuroimaging studies aimed at detecting group-level effects. Although a large number of neuroimaging studies have recognized structural brain abnormalities in depression, the patterns of abnormality remain unclear, as the findings have been largely inconsistent^41,44^. Patients with depression demonstrate decreased, no variation, or even increased gray matter volumes in distributed brain regions^45–48^. Among the affected brain regions, the hippocampus and medial prefrontal cortex are representative regions. Their structural differences are varied, resulting from the heterogeneity of patients with depression^12,49^. Collectively, our proposed framework helped parse subject-level morphological differences.

Although a number of studies also obtain subject-level neuroimaging differences, their results are difficult to understand^18,50,51^. The current study shows that group-level approaches only detect decreased gray matter volumes in limited brain regions, and the derived conclusions are representative of the “average” patient. Most of the individualized differences (e.g., positive factors) are concealed by group-level analysis. In this study, the identified disease factors show quantitative and linear relationship with group-level and subject-level differences, establishing a connection to previous findings.

Despite these advantages, this study also has some limitations. First, we don’t record enough clinical data, future studies can investigate whether disease factors correspond to symptom dimensions in depression. Second, only patients without other mental disorders comorbidities are included in this study. The influence of comorbidity on the disease factors can be investigated in the future. Third, we don’t control the alcohol and tobacco use.

This study proposes a robust and comprehensible framework to quantitatively estimate altered structural brain heterogeneity in depression from a dimensional perspective. Our proposed framework yields comprehensible neuroimaging results at subject level, reconciles the subject- and group-level neuroimaging findings and can predict factor compositions of patients in another independent dataset.

## Methods

### Sample

This study included three independent datasets: a discovery dataset and two validation datasets. The discovery dataset included 105 first-episode and untreated patients with depression and 130 matched healthy controls (HCs). Patients were recruited from the outpatient services of the Department of Psychiatry at the First Affiliated Hospital of Zhengzhou University. The protocol to recruit this dataset was approved by the Research Ethics Committee of the First Affiliated Hospital of Zhengzhou University. The validation dataset 1 included 76 patients with depression and 68 HCs. The protocol to recruit the validation dataset 1 was approved by the Research Ethics Committee of the University of Electronic Science and Technology of China. The validation dataset 2 included 492 HCs and the protocol to recruit this validation dataset was approved by the Research Ethics Committee of the Brain Imaging Center of Southwest University.

All study procedures were performed in accordance with the Helsinki Declaration of 1975, and written informed consent was obtained from all participants before the experiment. Details regarding the description of datasets and scan acquisition are included in the supplementary methods. All reported results were based on the discovery dataset and validated using the validation datasets.

### Voxel-based morphometry analysis

Voxel-wise gray matter volume (GMV) was measured following the recommended pipeline of the Computational Anatomy Toolbox (CAT12, http://dbm.neuro.uni-jena.de/cat12/). Structural images were segmented into gray and white matter and cerebrospinal fluid, normalized into Montreal Neurological Institute space (MNI), resampled to 1.5 mm^3^, smoothed (6-mm full-width at half-maximum Gaussian kernel)^38,52^. The total intracranial volume (TIV) of each participant was calculated. To control the data quality, the Image Quality Rating (IQR) was recorded^51,53^.

### Modelling latent disease factors

First, we adopted a normative model to obtain voxel-wise morphological differences at the subject level. In a similar manner to growth charts, which infer a child’s height or weight from age, the normative model infers subject-level neuroimaging differences. As in previous studies^8,18^, a Gaussian process regression was built to infer GMV value for each voxel from age and sex. The model was first trained with HCs and then applied to patients with depression. Two strategies were adopted to assess the performance of the normative model. Firstly, we assessed the performance of normative model using 10-fold cross-validation in HCs in three datasets, respectively^18^. Specifically, for each run, models were trained using the training set and predicted GMV values in the test set. Second, we trained a Gaussian process regression based on HCs in the discovery dataset and applied it to HCs in the validation dataset 1. The performance of models was assessed using the standardized mean squared error (MSE)^18^. Deviations between the predicted and true GMVs was measured with *Z*-score where positive *Z*-scores indicated higher GMV in patients, and vice versa. Voxels were divided into two parts according to *Z*-scores (positive and negative parts).

The subsequent steps were separately conducted on the positive and negative parts. NMF was conducted to parse individualized morphological differences into distinct patterns (disease factors) for the increased/decreased parts. As NMF only allows non-negative input, absolute values of *Z*-scores of negative parts were considered. In neuroimaging studies, NMF produces more explanatory, reproducible, and specific results and avoids the opposite differences canceling each other^54^. NMF yields soft (probabilistic) parcellation of voxel-wise differences. This method has been widely used in neuroimaging studies^55,56^. NMF is defined as follows:

2 $${{{{{\rm{D}}}}}}={{{{{\rm{W}}}}}}\times {{{{{\rm{H}}}}}}+{{{{{\rm{\epsilon }}}}}}{{{{{\rm{W}}}}}},{{{{{\rm{H}}}}}} > 0$$

Where D is the subject-level gray matter differences (subject × voxel), H is disease factors (factors × voxel), W (subject × K) is factor composition, and є is the residuals. The only parameter that needs to be predefined is the K.

Inspired by a previous study^57^, we determined the optimal K (between 2 and 10) using the following strategy. First, we assumed that if the K was the optimal, the corresponding results were the most stable. For each K (between 2 to 10), NMF was repeatedly ran because of random initialization (100 times). The Hungarian matching algorithm was used to match factors across runs^28,57^. The mean Pearson’s correlations between factors were calculated. This procedure yielded a 100 × 100 correlation matrix. The averaged value (stability value) of the 100 × 100 matrix was then calculated to measure the stability of the results across runs. If the K is optimal, the corresponding stability value was the largest. Finally, using the optimal K, we averaged the results across 100 runs. For each patient, linear regression models were generated to obtain how much variance was explained by disease factors.

Then, we investigated whether NMF with the optimal K would yield more stable disease factors and whether the variance explained by these disease factors was significantly greater than chance. To this end, a permutation test (1000 times) was performed. For each run, individualized GMV differences were shuffled for each patient. Then, we calculated stability value with the optimal K (as before). Once again, the variance of disease factors was explained using linear regression models. The significance was determined with permutation *p* < 0.05 (FDR corrected).

### Functional annotation for the disease factors

To provide a further interpretation of the identified disease factors, we conducted functional annotation on the identified disease factors. Briefly, functional annotation associated cognitive processes/functional terms with identified brain regions using probabilistic (activation) mappings provided by Neurosynth (https://neurosynth.org/)^24^. There are more than 4000 search terms in Neurosynth. However, some of the search terms were useless in identifying tasks such as “able” and “abstract.” Overall, 217 terms that had clear biological significance were selected. The list of selected functional terms is listed in the study by Cheng et al.^58^. The significance of the associations between the identified disease factors and functional terms was determined using a permutation test (1000 times). The significant terms were determined if the corresponding *p* values were <0.05 (FDR corrected). Details about the permutation test are included in the Supplementary Methods. As each factor covered the whole brain, functional annotation was performed on the most representative brain areas (the top 5% of voxels according to disease factors or H) for each factor. This procedure was performed using the brain annotation toolbox (BAT, https://istbi.fudan.edu.cn/lnen/info/1173/1788.htm)^59^.

### Association between factor composition and clinical features

We calculated Spearman’s correlation between factor composition and the Hamilton Depression Rating Scale factor scores, disease duration, age of onset, and years of education. The correlation results were corrected by false discovery rate (*p* < 0.05). The differences in factor composition between adolescent- (age ≤18 years) and adult-onset patients (age >18 years) were calculated^60^. Sexual differences in factor composition were also investigated using a two-sample *t* test (*p* < 0.05, FDR corrected).

### Robust analysis

To rule out the possibility that our results were biased by some patients, disease factors were identified from 90% of randomly selected patients with K determined in the previous step. This procedure was repeated 100 times. Spearman’s correlation coefficients between factor composition obtained from all patients and that from patient subgroup were calculated. Similarly, factors were matched with the Hungarian matching algorithm^28,57^.

### Reproducibility analysis

Then, we inferred the factor composition of patients in the validation dataset 1 using linear regression models where disease factors (identified using the discovery dataset) were the independent variables and subject-level differences were the dependent variables. The coefficients of regression models were treated as the predicted factor compositions. The true factor compositions of patients in the validation dataset 1 were obtained as before. Spearman’s correlation coefficients between the predicted factor composition and the true ones were calculated to measure the reproducibility of our framework. The correspondence between the predicted factor composition and the true ones were determined using the Hungarian matching algorithm.

### Association with group-level differences

We also investigated the relationship between subject- and group-level gray matter morphological differences. Group-level gray matter morphological differences were obtained by comparing GMV between all patients and HCs using a two-sample *t* test with using SPM12 software (http://www.fil.ion.ucl.ac.uk/spm). In this step age, sex, years of education and TIV were treated as covariates. Then, a linear regression model was conducted to assess the quantitative relationship between group-level gray matter morphological differences (unthresholded T map) and disease factors. Model statistics included the R^2^ statistic, the F-statistic and its *p*-value were recorded.

### Reporting summary

Further information on research design is available in the Nature Portfolio Reporting Summary linked to this article.

## Supplementary information

Supplementary Information

Reporting Summary

## Data Availability

The probabilistic (activation) mappings are provided by Neurosynth (https://neurosynth.org/). The neuroimaging data of discovery dataset and validation dataset 1 used in this study are under active use by the reporting laboratory and are available from the corresponding author upon request. The validation dataset 2 is freely available (http://fcon_1000.projects.nitrc.org/indi/retro/sald.html). Source data are provided with this paper.

## Source data

Source Data

## Code availability

All analytical procedures in this study are based on publicly available toolkits. Gray matter volume is assessed with the Computational Anatomy Toolbox (CAT12, http://dbm.neuro.uni-jena.de/cat12/). The normative model is freely available (GAUSSIAN PROCESS REGRESSION AND CLASSIFICATION Toolbox version 4.2, http://www.GaussianProcess.org/gpml/code). Functional annotation is performed using the Brain Annotation Toolbox (BAT version 1.1, https://istbi.fudan.edu.cn/lnen/info/1173/1788.htm). Group-level differences in GMV are obtained using SPM12 (http://www.fil.ion.ucl.ac.uk/spm). Non-negative matrix factorization is performed using the built-in function in MATLAB 2018a. Additional information is available from the corresponding author upon request.

## References

[1] Bondar J, Caye A, Chekroud AM, Kieling C (2020). Symptom clusters in adolescent depression and differential response to treatment: a secondary analysis of the treatment for adolescents with depression study randomised trial. Lancet Psychiatry.

[2] Krishnan V, Nestler EJ (2008). The molecular neurobiology of depression. Nature.

[3] Drysdale A. T., Grosenick L., Downar J. Resting-state connectivity biomarkers define neurophysiological subtypes of depression. **23**, 28–38 (2017). 10.1038/nm.4246 PMC5624035 27918562

[4] Nguyen T. D., Harder A., Xiong Y., Kowalec K., Hägg S. Genetic heterogeneity and subtypes of major depression. **27**, 1667–1675 (2022). 10.1038/s41380-021-01413-6 PMC9106834 34997191

[5] Goldberg D (2011). The heterogeneity of “major depression”. World Psychiatry.: Off. J. World Psychiatr. Assoc. (WPA).

[6] Asberg M, Bertilsson L, Tuck D, Cronholm B, Sjöqvist F (1973). Indoleamine metabolites in the cerebrospinal fluid of depressed patients before and during treatment with nortriptyline. Clin. Pharmacol. Ther..

[7] MacQueen G (2017). Systematic review of clinical practice guidelines for failed antidepressant treatment response in major depressive disorder, dysthymia, and subthreshold depression in adults. Can. J. Psychiatry Rev. Can. de. Psychiatr..

[8] Wolfers T (2018). Mapping the heterogeneous phenotype of schizophrenia and bipolar disorder using normative models. JAMA Psychiatry.

[9] Williams LM (2016). Precision psychiatry: a neural circuit taxonomy for depression and anxiety. Lancet Psychiatry.

[10] Chen Z (2016). High-field magnetic resonance imaging of structural alterations in first-episode, drug-naive patients with major depressive disorder. Transl. Psychiatry.

[11] Lynch CJ, Gunning FM, Liston C (2020). Causes and consequences of diagnostic heterogeneity in depression: paths to discovering novel biological depression subtypes. Biol. Psychiatry.

[12] Beijers L, Wardenaar KJ, van Loo HM, Schoevers RA (2019). Data-driven biological subtypes of depression: systematic review of biological approaches to depression subtyping. Mol. Psychiatry.

[13] Dunlop BW (2017). Functional connectivity of the subcallosal cingulate cortex and differential outcomes to treatment with cognitive-behavioral therapy or antidepressant medication for major depressive disorder. Am. J. Psychiatry.

[14] Collins FS, Varmus H (2015). A new initiative on precision medicine. N. Engl. J. Med..

[15] Hasler G (2010). Pathophysiology of depression: do we have any solid evidence of interest to clinicians?. World Psychiatry.: Off. J. World Psychiatr. Assoc. (WPA).

[16] Lahey BB, Pelham WE, Loney J, Lee SS, Willcutt E (2005). Instability of the DSM-IV Subtypes of ADHD from preschool through elementary school. Arch. Gen. Psychiatry.

[17] van Hulst BM, de Zeeuw P, Durston S (2015). Distinct neuropsychological profiles within ADHD: a latent class analysis of cognitive control, reward sensitivity and timing. Psychol. Med..

[18] Marquand AF, Rezek I, Buitelaar J, Beckmann CF (2016). Understanding heterogeneity in clinical cohorts using normative models: beyond case-control studies. Biol. Psychiatry.

[19] Wolfers T., Beckmann C. F. Individual differences v. the average patient: mapping the heterogeneity in ADHD using normative models. **50**, 314–323 (2020). 10.1017/S0033291719000084 PMC7083555 30782224

[20] Zabihi M (2019). Dissecting the heterogeneous cortical anatomy of autism spectrum disorder using normative models. Biol. Psychiatry Cogn. Neurosci. Neuroimaging.

[21] Ashburner J, Friston KJ (2000). Voxel-based morphometry–the methods. NeuroImage.

[22] Anderson A (2014). Non-negative matrix factorization of multimodal MRI, fMRI and phenotypic data reveals differential changes in default mode subnetworks in ADHD. NeuroImage.

[23] Shan X (2022). Mapping the heterogeneous brain structural phenotype of autism spectrum disorder using the normative model. Biol. Psychiatry.

[24] Yarkoni T, Poldrack RA, Nichols TE, Van Essen DC, Wager TD (2011). Large-scale automated synthesis of human functional neuroimaging data. Nat. Methods.

[25] Laurens VDM, Hinton G (2008). Visualizing data using t-SNE. J. Mach. Learn. Res..

[26] Chen H., et al. Parsing brain structural heterogeneity in males with autism spectrum disorder reveals distinct clinical subtypes. **40**, 628–637 (2019). 10.1002/hbm.24400 PMC6865602 30251763

[27] Chen H., et al. Dimensional analysis of atypical functional connectivity of major depression disorder and bipolar disorder. *Cerebral cortex* (New York, NY: 1991) **32**, 1307–1317 (2022). 10.1093/cercor/bhab296 34416760

[28] Zhang X (2016). Bayesian model reveals latent atrophy factors with dissociable cognitive trajectories in Alzheimer’s disease. Proc. Natl. Acad. Sci. USA.

[29] Han S., et al. Mapping the neuroanatomical heterogeneity of OCD using a framework integrating normative model and non-negative matrix factorization. *Cerebral cortex**(**New York, NY**:**1991*), (2023). 10.1093/cercor/bhad149 PMC10321110 37150510

[30] Harvey PO, Pruessner J, Czechowska Y, Lepage M (2007). Individual differences in trait anhedonia: a structural and functional magnetic resonance imaging study in non-clinical subjects. Mol. Psychiatry.

[31] Whitton AE, Treadway MT, Pizzagalli DA (2015). Reward processing dysfunction in major depression, bipolar disorder and schizophrenia. Curr. Opin. Psychiatry.

[32] Han S (2020). The anhedonia is differently modulated by structural covariance network of NAc in bipolar disorder and major depressive disorder. Prog. Neuro-Psychopharmacol. Biol. Psychiatry.

[33] Herzog DP (2021). Early onset of depression and treatment outcome in patients with major depressive disorder. J. Psychiatr. Res.

[34] Murphy CF (2007). White-matter integrity predicts stroop performance in patients with geriatric depression. Biol. Psychiatry.

[35] Bukh JD, Bock C, Vinberg M, Gether U, Kessing LV (2011). Differences between early and late onset adult depression. Clin. Pract. Epidemiol. Ment. Health.: CP EMH.

[36] Yu, M. et al. Childhood trauma history is linked to abnormal brain connectivity in major depression. *Proc. Natl Acad. Sci. USA* **116**, 8582–8590 (2019). 10.1073/pnas.1900801116 PMC6486762 30962366

[37] Kessler RC (2003). The epidemiology of major depressive disorder: results from the National Comorbidity Survey Replication (NCS-R). JAMA.

[38] Han, S. et al. Progressive brain structural abnormality in depression assessed with MR imaging by using causal network analysis. *Psychol Med*. **53**, 2146–2155 (2023). 10.1017/S0033291721003986 34583785

[39] Han S (2021). The stage-specifically accelerated brain aging in never-treated first-episode patients with depression. Hum. Brain Mapp..

[40] Mai N (2021). Different modular organization between early onset and late onset depression: a study base on granger causality analysis. Front. Aging Neurosci..

[41] Schmaal L., et al. Subcortical brain alterations in major depressive disorder: findings from the ENIGMA major depressive disorder working group. **21**, 806–812 (2016). 10.1038/mp.2015.69 PMC4879183 26122586

[42] Blank T. S., Meyer B. M. Brain morphometry and connectivity differs between adolescent- and adult-onset major depressive disorder. **39**, 387–396 (2022). 10.1002/da.23254 PMC9323432 35421280

[43] Nickson T (2016). Prospective longitudinal voxel-based morphometry study of major depressive disorder in young individuals at high familial risk. Psychol. Med..

[44] Lorenzetti V, Allen NB, Fornito A, Yücel M (2009). Structural brain abnormalities in major depressive disorder: a selective review of recent MRI studies. J. Affect. Disord..

[45] Wise T., Radua J., Via E., Cardoner N., Abe O. Common and distinct patterns of grey-matter volume alteration in major depression and bipolar disorder: evidence from voxel-based meta-analysis. **22**, 1455–1463 (2017). 10.1038/mp.2016.72 PMC5622121 27217146

[46] McKinnon MC, Yucel K, Nazarov A, MacQueen GM (2009). A meta-analysis examining clinical predictors of hippocampal volume in patients with major depressive disorder. J. Psychiatry Neurosci.: JPN.

[47] Yang X (2015). Gray matter volume abnormalities were associated with sustained attention in unmedicated major depression. Compr. Psychiatry.

[48] Zhang X (2012). Gray matter volume abnormalities in individuals with cognitive vulnerability to depression: a voxel-based morphometry study. J. Affect. Disord..

[49] Belleau EL, Treadway MT, Pizzagalli DA (2018). The impact of stress and major depressive disorder on hippocampal and medial prefrontal cortex morphology. Biol. Psychiatry.

[50] Han, S. et al. Resolving heterogeneity in depression using individualized structural covariance network analysis. *Psychol. Med.*10.1017/S0033291722002380, 1–10 (2022). 10.1017/S0033291722002380 35959558

[51] Han S., et al. Resolving heterogeneity in obsessive-compulsive disorder through individualized differential structural covariance network analysis. *Cerebral cortex**(**New York, NY**:**1991*), (2022). 10.1093/cercor/bhac163 35470393

[52] Ashburner J (2009). Computational anatomy with the SPM software. Magn. Reson. Imaging.

[53] Brown JA (2019). Patient-tailored, connectivity-based forecasts of spreading brain atrophy. Neuron.

[54] Sotiras A (2017). Patterns of coordinated cortical remodeling during adolescence and their associations with functional specialization and evolutionary expansion. Proc. Natl. Acad. Sci. USA.

[55] Li H, Satterthwaite TD, Fan Y (2017). Large-scale sparse functional networks from resting state fMRI. NeuroImage.

[56] Sotiras A, Resnick SM, Davatzikos C (2015). Finding imaging patterns of structural covariance via Non-Negative Matrix Factorization. NeuroImage.

[57] Lee H. M., et al. Decomposing MRI phenotypic heterogeneity in epilepsy: a step towards personalized classification. **145**, 897–908 (2022). 10.1093/brain/awab425 PMC9050524 34849619

[58] Cheng W (2017). Functional connectivity decreases in autism in emotion, self, and face circuits identified by Knowledge-based Enrichment Analysis. NeuroImage.

[59] Liu Z (2019). Brain annotation toolbox: exploring the functional and genetic associations of neuroimaging results. Bioinforma. (Oxf., Engl.).

[60] Harald B, Gordon P (2012). Meta-review of depressive subtyping models. J. Affect. Disord..

