# Supplementary material for: Parsing altered gray matter morphology of depression using a framework integrating the normative model and non-negative matrix factorization
Source: Nat Commun. 2023 Jul 8;14:4053. doi: 10.1038/s41467-023-39861-z (PMC10329663; doi:10.1038/s41467-023-39861-z)
Supplement: Supplementary file 1 — Supplementary Information [file 41467_2023_39861_MOESM1_ESM.pdf]

Supplementary results for
Parsing altered gray matter morphology of depression using a framework integrating the normative model and non-negative matrix factorization

Table of Contents

Supplementary Methods 2

Datasets 2

Data acquisition 3

Functional annotation for the disease factors 4

Supplementary results 4

Permutation test results of NMF 4

The group-level differences can be derived from the identified disease factors in the validation dataset 4

Table S1. Functional annotation results for the identified disease factors in the discovery dataset 5

Table S2. Functional annotation results for the identified disease factors in the validation dataset 1 7

Figure S1. Stability values of different number of disease factors in the discovery dataset and validation dataset 1. 9

Figure S2. Functional annotation results of the identified disease factors in the validation dataset 1. 10

Figure S3. Functional annotation results of the identified disease factors in the discovery dataset. 11

Figure S4. Group-level gray matter morphological differences. 12

Figure S5. The standardized mean squared error (MSE) for the normative model. 13

Supplementary Methods

Datasets

Discovery dataset

105 first-episode and untreated patients with depression were recruited from out-patient services of Department of Psychiatry, the First Affiliated Hospital of Zhengzhou University. Patients were diagnosed according to the Diagnostic and Statistical Manual of Mental Disorders, Fourth Edition (DSM-IV) for depression. The diagnosis was done by one chief physician and one trained psychiatrist. None of patients displayed comorbidity with other mental (psychotic) disorders or a history of mania. All patients were in depressed phase. The clinical states were evaluated using the 17-items Hamilton Depression scale (HAMD). According to previous studies, the HAMD scores were further divided into five factors: psychomotor slowing (including depressed mood; loss of interest in activity, hobbies or work; slowness of thought and speech, impaired ability to concentrate, decreased motor activity and sexual symptoms), cognitive impairment (including feelings of guilt; suicide; and agitated behavior), anxiety/somatization (including psychic anxiety; somatic anxiety; gastro-intestinal symptoms; general somatic symptoms; hypochondriasis; insight), sleep disturbance (including difficulty in falling asleep; light sleep and early awakening), and weight [1, 2, 3].The healthy controls were interviewed using for DSM-IV Non-Patient version (SCID-NP). We have added this in new version. Healthy controls (HCs) (n = 130) were recruited from the community through online platforms and advertisements. The healthy controls were interviewed using for DSM-IV Non-Patient version (SCID-NP). All patients and HCs were Han Chinese and right handedness.

For all participants, additional exclusion criteria included: (1) Taking medicines such as analgesia, sleeping in the past month; (2) A history of substance abuse; (3) A history of trauma, brain surgery, or other organic body disease affection the anatomical structures of the brain; (4) A history of diabetes, hypertension or cardiovascular diseases; (5) MRI scanning contraindications; (6) Other structural brain abnormalities revealed by MRI scan.

First, patients with depression were recruited. Followed by the HCs to ensure demographic characteristics did not significantly differ from patients. Only participant who met the inclusion and exclusion criteria were scanned. Once the scan was over, an experienced scanning technician would check the image quality. For each subject, if its image quality was poor or the scan contained artifacts, it would be repeated. Some subjects were excluded for structural brain abnormalities, and these subjects who were not willing to be rescanned were excluded. When we collected clinical information and images, these subjects were not counted.

Validation dataset 1

The replication data included 76 patients with depression and matched 68 matched HCs. All patients with depression were interviewed by two experienced psychiatrists using the Structured Clinical Interview for DSM-IV-TR-Patient Edition (SCID-P, 2/2001 revision) for depression. The 24-Item

Hamilton Depression Scale was used to evaluate the clinical state of the patients and all patients were under depressive state. Patients were excluded if they met one of the exclusion criteria: schizophrenia, mental retardation, personality disorder, any history of loss of consciousness, substance abuse, and serious medical or neurological illness. Patients were excluded if they met the diagnostic criteria for anxiety. Patients with depression were treated with antidepressants. The drugs administered included one of the selective serotonin and serotonin–norepinephrine reuptake inhibitors. HCs were recruited from the community through poster advertisements and interviewed using SCID (nonpatient edition). None of HCs had a history of serious medical or neuropsychiatric illness and a family history of major psychiatric or neurological illness in their first-degree relatives.

Validation dataset 2

The validation dataset 2 included 494 healthy subjects (308 Females, 187 Males, aged 19 to 80 yeas) from Southwest University Adult Lifespan Dataset (SALD) study freely available (http://fcon_1000.projects.nitrc.org/indi/s3/index.html). The exclusion criteria included MRI-related exclusion criteria, current psychiatric/neurological disorders, use of psychiatric drugs in the past three months prior to scanning, pregnancy and a history of head trauma. More detailed description about the subject information, please see [4]. Please note, we exclude two females for poor image quality in this study.

Data acquisition

Discovery dataset

Using an 8-channel prototype quadrature birdcage head coil, T1-weighted anatomical images with 3D spoiled gradient echo scan sequence were acquired on 3-Tsela GE Discovery MR750 scanner (General Electric, Fairfield Connecticut, USA). The scanning parameters were as followed: voxel size = $1 \times 1 \times 1$ mm^3, repetition time = 8,164 ms, flip angle = 7 degrees, inversion time = 900 ms, echo time = 3.18 ms, thickness = 1.0 mm, resolution matrix = 256×256, slices = 188.

Validation dataset 1

The validation dataset 1 was acquired on a 3-Tesla GE Discovery MR750 scanner. Structural T1-weighted images with 3D spoiled gradient echo scan sequence with the following parameters: TR/TE = 5.92/1.956 ms, voxel size = 1 mm × 1 mm × 1 mm, slice thickness = 1 mm, no gap, flip angle = 12 degrees, matrix size = 256×256, and 156 slices.

Validation dataset 2

The validation dataset 2 was acquired on a 3.0-T Siemens Trio MRI scanner (Siemens Medical, Erlangen, Germany) using a magnetization-prepared rapid gradient echo (MPRAGE) sequence with the following parameters: repetition time = 1,900 ms, echo time = 2.52 ms, flip angle = 90 degrees, inversion time = 900 ms, resolution matrix = 256×256, slices = 176, thickness = 1.0 mm, and voxel

size = 1 × 1 × 1 mm^3.

Functional annotation for the disease factors

We just briefly described the main steps about functional annotation, more details were included in [5]. Similar to gene enrichment analysis, in neuroimaging studies, voxels within a cluster had a higher probability to be co-activated by the same functional terms than voxels randomly selected. For a functional term, the activation ratio (ACR) was used to measure the extent of activation of a given cluster. The definition of ACR was as follow:

$$\mathrm{ACR_i} = \frac{\mathrm{x_i}}{\mathrm{N_i}} \quad (1)$$

Where N_i was the number of voxels in the region i. x_i was the number of voxels activated in the region i for the given functional term.

The significance of the ACR was assessed using 1000 times permutation test. For each run, we randomly selected the same number (as those in the given region) of voxels from the background calculated ACR_rand. Then, p value was the ratio of ACR_rand values greater than the true ACR.

Supplementary results

Permutation test results of NMF

Permutation test results suggested that NMF yielded significantly greater stable values (permutation $p < 0.001$) and explained greater variance than chance (FDR corrected permutation p values < 0.001). Each disease factors demonstrated significant association with individualized morphological differences (PF1: 0.13 ± 0.08, PF2: 0.10 ± 0.08, NF1: 0.13 ± 0.07, NF2: 0.10 ± 0.08, FDR corrected permutation p values < 0.01).

The group-level differences can be derived from the identified disease factors in the validation dataset

In validation dataset, we also found that the group-level differences (unthresholded T map) could be significantly derived from the identified 4 disease factors ($R^2 = 0.728$, $p < 0.001$, $F = 2.239\times10^5$). The association between group-level differences and the identified 4 disease factors was:

$$\text{Group-level differences} = 0.824^3 * \text{factor}_{p1} + 0.621\times10^3 * \text{factor}_{p2} - 0.630\times10^3 * \text{factor}_{n1} - 0.841\times10^3 * \text{factor}_{n2} \quad (2)$$

Table S1. Functional annotation results for the identified disease factors in the discovery dataset

Disease factors	Functional terms	FDR corrected *p*	Disease factors	Functional terms	FDR corrected *p*
PF1	music	0.026	NF1	affective	0.020
	acoustic	0.046		anticipation	0.018
	autobiographical_memory	0.010		anxiety	0.025
	language_comprehension	0.044		arousal	0.009
	language_network	0.046		bipolar_disorder	0.040
	language	0.045		cognitive_emotional	0.038
	listening	0.049		compulsive_disorder	0.039
	phonetic	0.038		conscious	0.005
	semantic_knowledge	0.047		decision_making	0.030
	semantic	0.026		emotion	0.034
	sentence_comprehension	0.022		emotional_responses	0.030
	social_cognition	0.038		fear_pAgF	0.025
	speech	0.049		gambling	0.045
	speech_perception	0.037		hyperactivity_disorder	0.028
	speech_sounds	0.029		impulsivity	0.037
	spoken	0.022		incentive	0.022
	syntactic	0.019		monetary_reward	0.033
	theory_mind	0.044		mood	0.046
PF2	voice	0.027		motivation	0.019
	affective	0.015		motor_response	0.042
	anger	0.006		neutral_pictures	0.011
	anticipation	0.010		nociceptive	0.004
	anxiety_disorders	0.002		obsessive_compulsive	0.037
	anxiety	0.002		ongoing	0.036
	arousal	0.003		pain	0.002
	attention_deficit	0.002		personality	0.045
	autism	0.028		pleasant	0.015
	bipolar_disorder	0.021		pressure	0.005
	cognitive_emotional	0.028		primary_somatosensory	0.036
	compulsive_disorder	0.049		reactivity	0.018
	conscious	0.029		reward	0.011
	decision_making	0.010		salience	0.009
	depression	<0.001		secondary_somatosensory	0.027
	disgust	0.009		self	0.025
	emotion	0.011		self_reported	0.001
	emotion_regulation	0.032		sensation	0.004
	emotional_faces	0.013		sleep	<0.001
	emotional_information	0.007		social_interaction	0.017
	emotional_responses	0.002		somatosensory	0.042
	emotional_stimuli	0.002		stress	0.025
	emotional_valence	0.004		sustained_attention	0.031
	encoding_retrieval	0.046		vulnerability	0.009
	face_stimuli	0.049	NF2	acoustic	0.002
	facial_expressions	0.033		auditory_visual	0.020

	fear	0.007		communication	0.001
	fearful_faces	0.018		hallucinations	0.049
	gain	0.009		language_comprehension	0.001
	gambling	0.005		language	0.024
	happy	0.004		listening	0.004
	hyperactivity_disorder	0.004		music	0.007
	impulsivity	0.025		perception	0.043
	incentive	0.011		phonetic	0.003
	learning_task	<0.001		primary_auditory	0.016
	major_depression	<0.001		speech	0.002
	memory_encoding	0.048		speech_perception	0.003
	mild_cognitive	0.011		speech_production	0.029
	monetary_reward	0.013		speech_sounds	0.004
	mood	0.001		spoken	0.002
	motivation	0.009		syntactic	0.047
	negative_emotional	<0.001		theory_mind	0.040
	neutral_faces	0.019		visual_auditory	0.046
	neutral_pictures	0.008		voice	0.001
	neutral_stimuli	0.006			
	passive_viewing	0.029			
	personality	0.003			
	pleasant	0.001			
	reactivity	<0.001			
	recognition_memory	0.044			
	reward_anticipation	0.007			
	reward	0.004			
	sad	0.001			
	salience	0.002			
	self	0.036			
	self_reported	0.002			
	sleep	0.037			
	social_interaction	0.025			
	stress	<0.001			
	subsequent_memory	0.040			
	threatening	0.019			
	vulnerability	<0.001			

Table S2. Functional annotation results for the identified disease factors in the validation dataset 1

Disease factors	Functional terms	FDR corrected *p*	Disease factors	Functional terms	FDR corrected *p*
PF1	fusiform_face	< 0.001	PF2	hyperactivity_disorder	0.001
	attention_deficit	0.001		multisensory	< 0.001
	awareness	< 0.001		music	0.001
	bipolar_disorder	0.001		primary_auditory	0.001
	conscious	0.001	NF1	anger	< 0.001
	hyperactivity_disorder	0.001		conscious	< 0.001
	suppression	< 0.001		disgust	< 0.001
	sustained_attention	0.001		emotional_faces	0.001
	attention	< 0.001		emotional_stimuli	0.001
	face	< 0.001		fear	< 0.001
	fusiform_face	0.001		fearful_faces	0.001
	memory_encoding	0.001		happy	0.001
	autobiographical_memeory	< 0.001		negative_emotional	0.001
	motion	< 0.001		neutral_faces	0.001
	naming	< 0.001		neutral_pictures	< 0.001
	object_recognition	< 0.001		neutral_stimuli	0.001
	oral	< 0.001		sad	< 0.001
	percept	< 0.001		phonetic	< 0.001
	perceptual	< 0.001		speech	< 0.001
	phonological	< 0.001		speech_sounds	0.001
	picture	< 0.001		voice	< 0.001
	reading	0.001		sleep	0.001
	recognition	< 0.001		sleep	0.001
	semantic	< 0.001		acoustic	< 0.001
	sensory_modalities	< 0.001		auditory_visual	0.001
	visual_attention	< 0.001		hallucinations	0.001
	visual_information	< 0.001		language	0.001
	visual_stimuli	< 0.001		listening	0.001
	visual_stream	0.001		music	< 0.001
	word_recognition	< 0.001		perception	< 0.001
	working_memory	< 0.001		perceptual	0.001
	written	< 0.001		phonological	< 0.001
	language	< 0.001		primary_auditory	< 0.001
	memory	< 0.001		speech	< 0.001
	phonological	< 0.001		speech_production	0.001
	reading	0.001		speech_sounds	0.001
	semantic	0.001		spoken	< 0.001
	syntactic	0.001		visual_auditory	0.001
	acoustic	< 0.001		voice	< 0.001
	multisensory	0.001		mental_imagery	< 0.001
	control_processes	< 0.001		decision_task	< 0.001

	decision_task	0.001		lexical_decision	< 0.001
	language	< 0.001		matching_task	< 0.001
	lexical_decision	0.001		memory_wm	< 0.001
	phonological	< 0.001		repetition_suppression	0.001
	stroop_task	< 0.001		suppression	0.001
	working_memory	0.001		verbal_working	< 0.001
PF2	anxiety_disorders	0.001		motor_performance	< 0.001
	autism	0.001		rhythm	< 0.001
	depression	< 0.001		tapping	< 0.001
	emotional_faces	0.001	NF2	motion	< 0.001
	emotional_information	0.001		multisensory	0.004
	emotional_stimuli	0.001		object_recognition	0.004
	emotional_valence	0.001		percept	0.004
	encoding_retrieval	0.001		perception	0.002
	episodic_memory	< 0.001		perceptual	< 0.001
	fearful_faces	0.001		primary_visual	< 0.001
	happy	< 0.001		visual_attention	0.001
	major_depression	0.001		visual_auditory	0.002
	memory_encoding	< 0.001		visual_information	0.001
	memory	0.001		visual_motion	0.003
	memory_performance	0.001		visual_perception	0.003
	mild_cognitive	0.001		visual_stimuli	< 0.001
	mood	< 0.001		visual_stream	0.004
	negative_emotional	< 0.001		language_comprehension	0.004
	neutral_pictures	0.001		language_network	< 0.001
	neutral_stimuli	< 0.001		language	0.002
	personality	0.001		reading	0.004
	pleasant	< 0.001		semantic_information	< 0.001
	reactivity	< 0.001		semantic	0.002
	recognition_task	0.001		phonological	0.003
	recollection	0.001		reading	0.004
	sad	< 0.001		attention	0.002
	stress	0.001		detection_task	0.001
	subsequent_memory	0.001		selective_attention	0.001
	threatening	< 0.001		visual_attention	0.002
	vulnerability	0.001		visual_stream	0.001
	face_recognition	0.001		visuo_spatial	0.002
	fusiform_face	0.001		hand_movements	0.004
	attention_deficit	0.001			

Figure S1. Stability values of different number of disease factors in the discovery dataset and validation dataset 1. Source data are provided as a Source Data file.

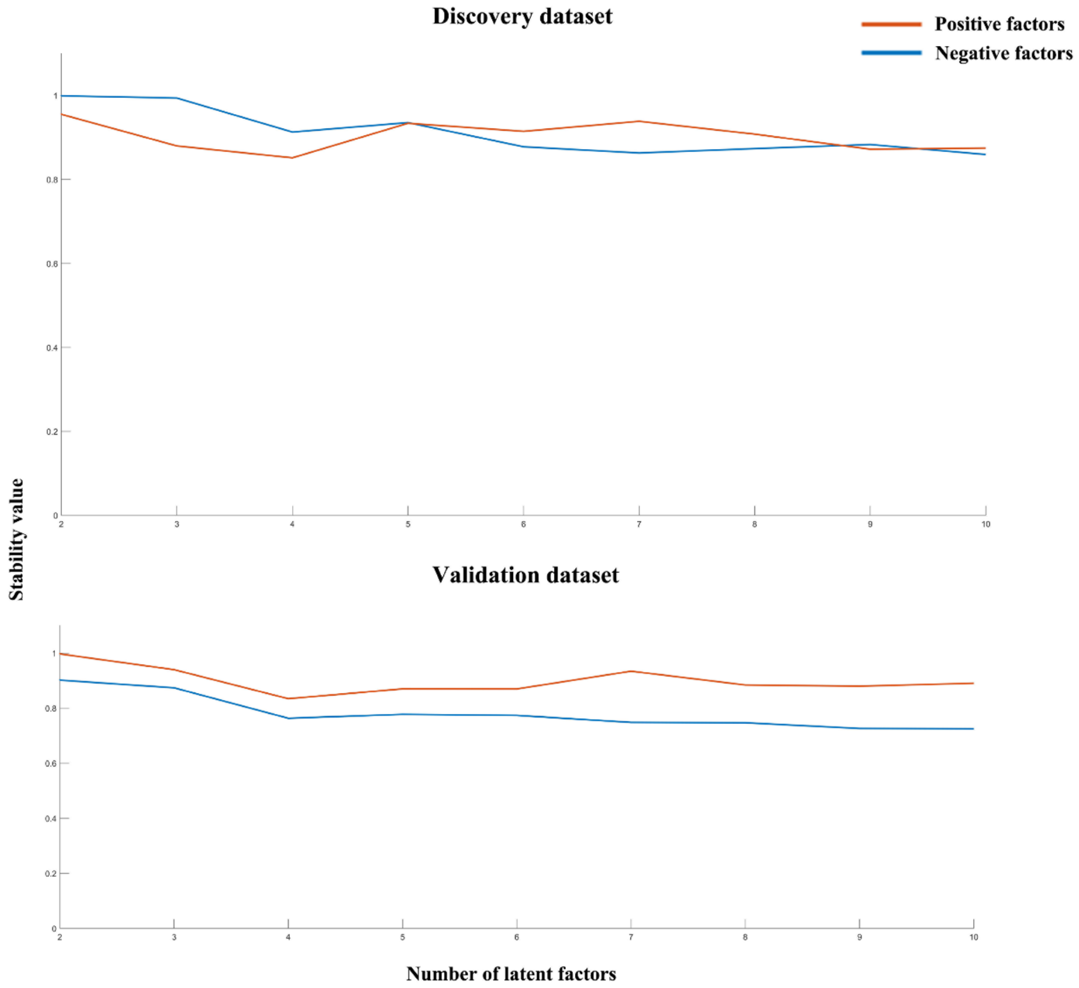

Figure S2. Functional annotation results of the identified disease factors in the validation dataset 1. The numbers beside the disease factors represents their spatial correlation with those in the discovery dataset (all p values < 0.001, FDR corrected). The size of word is proportional to $1/p$. Note, PF1, positive factor 1; PF2, positive factor 2; NF1, negative factor 1; NF2, negative factor 2. For each disease factor, the most representative functional terms are colored orange.

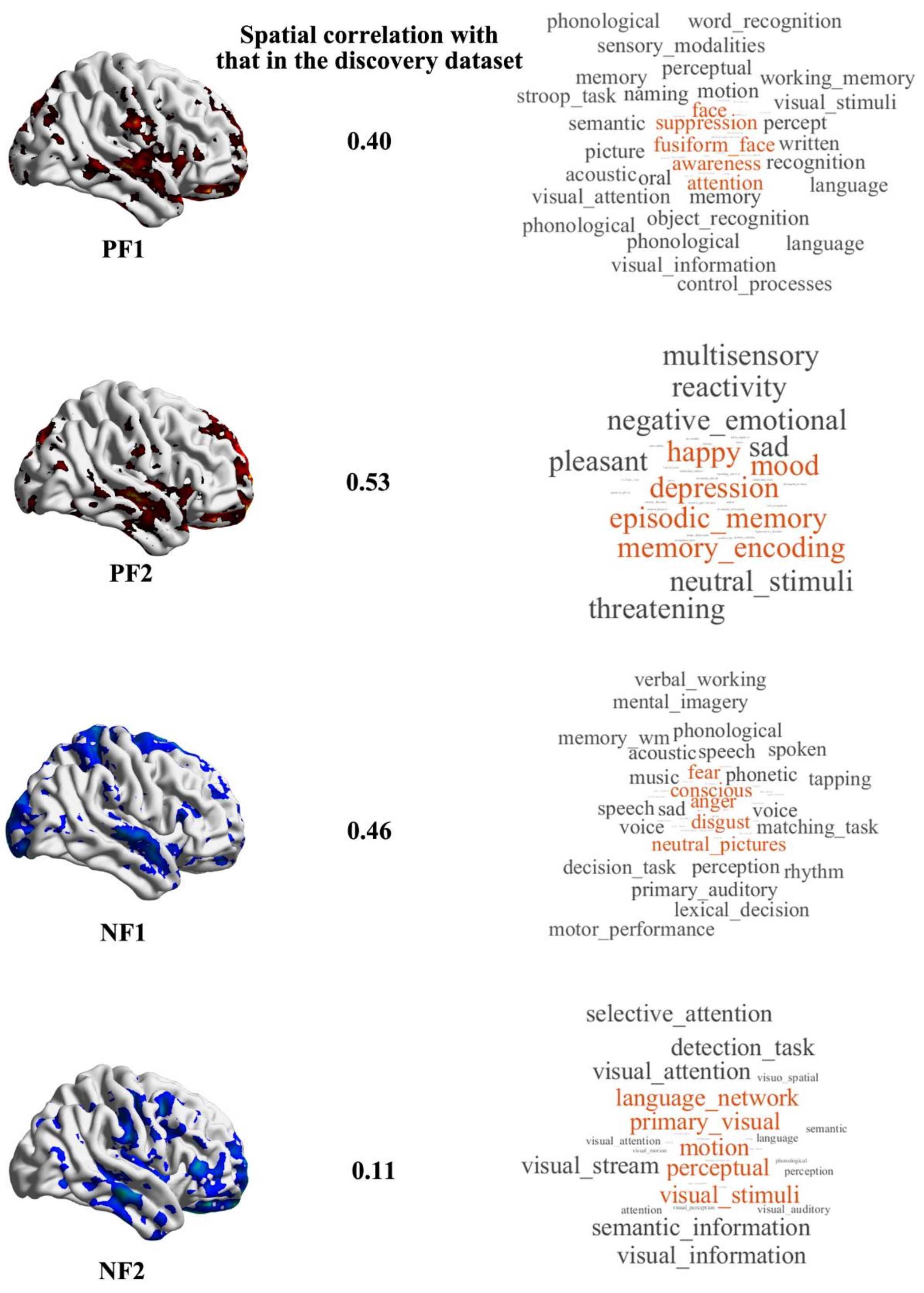

Figure S3. Functional annotation results of the identified disease factors in the discovery dataset. The size of word is proportional to 1/ *p*. Note, PF1, positive factor 1; PF2, positive factor 2; NF1, negative factor 1; NF2, negative factor 2. For each disease factor, the most representative functional terms are colored orange.

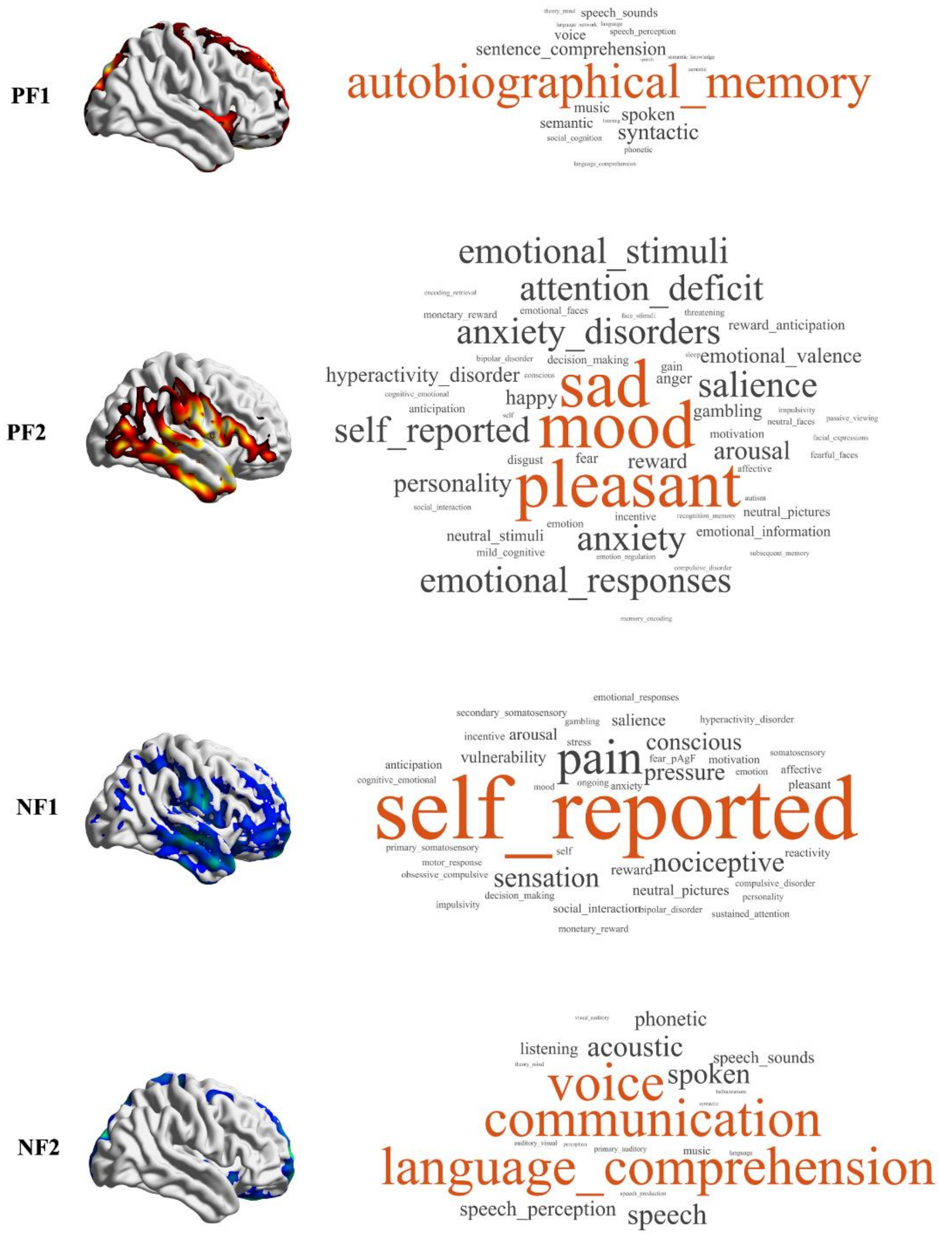

Figure S4. Group-level gray matter morphological differences (patients vs. healthy controls) obtained using two-sided two-sample *t* test ($p < 0.05$, FDR corrected) in discovery dataset.

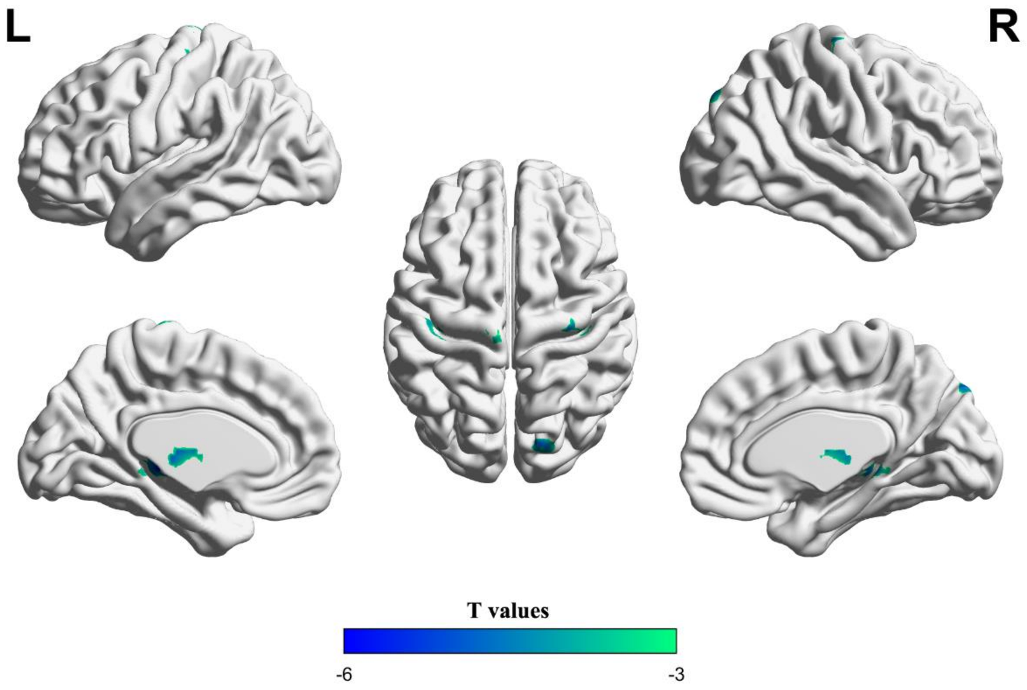

Figure S5. The standardized mean squared error (MSE) for the normative model under 10-fold cross-validation (averaged across all cross-validation folds) in healthy controls in three datasets. We also apply the normative model trained using discovery dataset to the validation dataset 1. We do not apply the trained model to validation dataset 2 for their different age distributions. These results suggest that voxel-wise gray matter volume can be accurately predicted across different datasets.

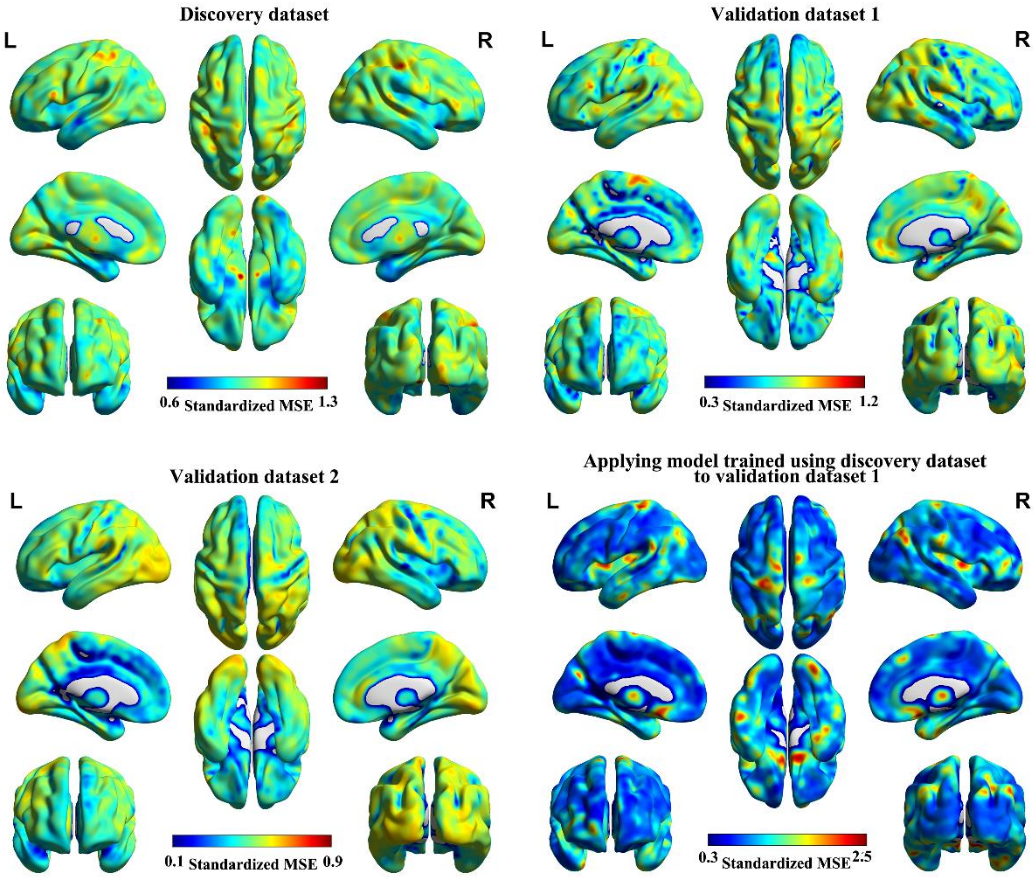

Supplementary References

1. Zhao B*, et al.* Can acupuncture combined with SSRIs improve clinical symptoms and quality of life in patients with depression? Secondary outcomes of a pragmatic randomized controlled trial. *Complementary therapies in medicine* **45**, 295-302 (2019).

2. Zhao S*, et al.* Sex differences in the association between symptom profiles and cognitive functioning in patients with depressive disorder. *Journal of affective disorders* **287**, 1-7 (2021).

3. Guo T*, et al.* Electroacupuncture and cognitive behavioural therapy for sub-syndromal depression among undergraduates: a controlled clinical trial. *Acupuncture in medicine : journal of the British Medical Acupuncture Society* **34**, 356-363 (2016).

4. Wei D*, et al.* Structural and functional brain scans from the cross-sectional Southwest University adult lifespan dataset. *Scientific data* **5**, 180134 (2018).

5. Liu Z*, et al.* Brain annotation toolbox: exploring the functional and genetic associations of neuroimaging results. *Bioinformatics (Oxford, England)* **35**, 3771-3778 (2019).
