# Supplementary material for: Parsing altered gray matter morphology of depression using a framework integrating the normative model and non-negative matrix factorization
Source: Nat Commun. 2023 Jul 8;14:4053. doi: 10.1038/s41467-023-39861-z (PMC10329663; doi:10.1038/s41467-023-39861-z)
Supplement: Supplementary file 2 — Reporting Summary [file 41467_2023_39861_MOESM2_ESM.pdf]

nature portfolio

Corresponding author(s): Shaoqiang Han, Yuan Chen, Jingliang Cheng, Huafu Chen

Last updated by author(s): May 22, 2023

Reporting Summary

Nature Portfolio wishes to improve the reproducibility of the work that we publish. This form provides structure for consistency and transparency in reporting. For further information on Nature Portfolio policies, see our Editorial Policies and the Editorial Policy Checklist.

Statistics

For all statistical analyses, confirm that the following items are present in the figure legend, table legend, main text, or Methods section.

n/a	Confirmed	
☐	☒	The exact sample size (n) for each experimental group/condition, given as a discrete number and unit of measurement
☐	☒	A statement on whether measurements were taken from distinct samples or whether the same sample was measured repeatedly
☐	☒	The statistical test(s) used AND whether they are one- or two-sided<br>*Only common tests should be described solely by name; describe more complex techniques in the Methods section.*
☐	☒	A description of all covariates tested
☐	☒	A description of any assumptions or corrections, such as tests of normality and adjustment for multiple comparisons
☐	☒	A full description of the statistical parameters including central tendency (e.g. means) or other basic estimates (e.g. regression coefficient) AND variation (e.g. standard deviation) or associated estimates of uncertainty (e.g. confidence intervals)
☐	☒	For null hypothesis testing, the test statistic (e.g. F, t, r) with confidence intervals, effect sizes, degrees of freedom and P value noted<br>*Give P values as exact values whenever suitable.*
☒	☐	For Bayesian analysis, information on the choice of priors and Markov chain Monte Carlo settings
☒	☐	For hierarchical and complex designs, identification of the appropriate level for tests and full reporting of outcomes
☐	☒	Estimates of effect sizes (e.g. Cohen's d, Pearson's r), indicating how they were calculated

Our web collection on statistics for biologists contains articles on many of the points above.

Software and code

Policy information about availability of computer code

Data collection	Structural MRI sanner (see Methods)
Data analysis	All analytical procedures in this study are based on publicly available toolkits. Gray matter volume is obtained using the Computational Anatomy Toolbox (CAT12, http://dbm.neuro.uni-jena.de/cat12/). The normative model is freely available (GAUSSIAN PROCESS REGRESSION AND CLASSIFICATION Toolbox version 4.2, http://www.GaussianProcess.org/gpml/code). Functional annotation is performed using the Brain Annotation Toolbox (BAT version 1.1, https://istbi.fudan.edu.cn/lnen/info/1173/1788.htm). Group-level differences in GMV are obtained using SPM12 (http://www.fil.ion.ucl.ac.uk/spm). Non-negative matrix factorization is performed using the built-in function in MATLAB 2018a. Additional information is available from the corresponding author upon reasonable request.

For manuscripts utilizing custom algorithms or software that are central to the research but not yet described in published literature, software must be made available to editors and reviewers. We strongly encourage code deposition in a community repository (e.g. GitHub). See the Nature Portfolio guidelines for submitting code & software for further information.

Data

Policy information about availability of data

All manuscripts must include a data availability statement. This statement should provide the following information, where applicable:

- Accession codes, unique identifiers, or web links for publicly available datasets
- A description of any restrictions on data availability
- For clinical datasets or third party data, please ensure that the statement adheres to our policy

The probabilistic (activation) mappings are provided by Neurosynth (https://neurosynth.org/). The neuroimaging data of discovery dataset and validation dataset 1 used in this study are protected and are not available due to data privacy laws and data use agreements. The validation dataset 2 is freely available (http://fcon_1000.projects.nitrc.org/indi/s3/index.html). Source data are provided with this paper.

Human research participants

Policy information about studies involving human research participants and Sex and Gender in Research.

Reporting on sex and gender	The gender (self-reporting) and sex were unified for all subjects in this study. All results were obtained based on both male and female patients.
Population characteristics	This study included three independent datasets: a discovery dataset and two validation datasets. The discovery dataset included 105 first-episode untreated patients (53 males, mean age ± SD=20.30 ± 5.04) with depression and 130 healthy controls (HCs, 59 males, mean age ± SD=21.05 ± 5.33) recruited from the outpatient services of the Department of Psychiatry at the First Affiliated Hospital of Zhengzhou University. The validation dataset 1 included 76 patients with depression (30 males, mean age ± SD=24.76 ± 6.58) and 68 HCs (35 males, mean age ± SD=25.06 ± 5.12). The validation dataset 2 included 492 HCs (187 males, mean age ± SD=45.10 ± 17.30).
Recruitment	In the discovery dataset, one hundred and five first-episode untreated patients with depression were recruited from out-patient services of Department of Psychiatry, the First Affiliated Hospital of Zhengzhou University. Patients were diagnosed by one chief physician and one trained psychiatrist according to the Diagnostic and Statistical Manual of Mental Disorders, Fourth Edition (DSM-IV) for depression. Healthy controls (HCs) (n = 130) were recruited from the community through poster advertisements. The healthy controls were interviewed using for DSM-IV Non-Patient version (SCID-NP). All patients and HCs were Han Chinese and right handedness. In addition, all participants must meet the following exclusion criteria: (1) Taking drugs such as anesthesia, sleeping and analgesia in the past month; (2) Substance abuse (or a history of substance abuse); (3) A history of brain tumor, trauma, surgery, or other organic body disease affection the anatomical structures of the brain; (4) Suffering from cardiovascular diseases, diabetes or hypertension; (5) Contraindications for MRI scanning; (6) Other structural brain abnormalities revealed by MRI scan.<br>In the validation dataset 1, All patients with depression were interviewed by two experienced psychiatrists using the Structured Clinical Interview for DSM-IV-TR-Patient Edition (SCID-P, 2/2001 revision) for depression. The 24-Item Hamilton Depression Scale was used to evaluate the clinical state of the patients and all patients were under depressive state. Patients were excluded if they met one of the exclusion criteria: schizophrenia, mental retardation, personality disorder, any history of loss of consciousness, substance abuse, and serious medical or neurological illness. Patients were excluded if they met the diagnostic criteria for anxiety. Patients with depression were treated with antidepressants. The drugs administered included one of the selective serotonin and serotonin–norepinephrine reuptake inhibitors. HCs were recruited from the community through poster advertisements and interviewed using SCID (nonpatient edition). None of HCs had a history of serious medical or neuropsychiatric illness and a family history of major psychiatric or neurological illness in their first-degree relatives.<br>The validation dataset 2 included 494 healthy subjects (308 Females, 187 Males, aged 19 to 80 yeas) from Southwest University Adult Lifespan Dataset (SALD) study freely available (http://fcon_1000.projects.nitrc.org/indi/s3/index.html). The exclusion criteria included MRI-related exclusion criteria, current psychiatric/neurological disorders, use of psychiatric drugs in the past three months prior to scanning, pregnancy and a history of head trauma.
Ethics oversight	The protocol to recruit the discovery dataset was approved by the Research Ethics Committee of the First Affiliated Hospital of Zhengzhou University. The protocol to recruit the validation dataset 1 was approved by the Research Ethics Committee of the University of Electronic Science and Technology of China.The protocol to recruit validation dataset 2 was approved by the Research Ethics Committee of the Brain Imaging Center of Southwest University.

Note that full information on the approval of the study protocol must also be provided in the manuscript.

Field-specific reporting

Please select the one below that is the best fit for your research. If you are not sure, read the appropriate sections before making your selection.

☒ Life sciences ☐ Behavioural & social sciences ☐ Ecological, evolutionary & environmental sciences

For a reference copy of the document with all sections, see nature.com/documents/nr-reporting-summary-flat.pdf

Life sciences study design

All studies must disclose on these points even when the disclosure is negative.

Sample size	Sample size was based on availability of data, no statistical methods were used to pre-determine sample size.
Data exclusions	(1) Taking drugs such as anesthesia, sleeping and analgesia in the past 1 month; (2) Substance abuse; (3) A history of brain tumor, trauma, surgery, or other organic body disease; (4) Suffering from cardiovascular diseases, diabetes or hypertension; (5) Contraindications for MRI scanning; (6) Other structural brain abnormalities revealed by MRI scan.
Replication	To rule out the possibility that the identified disease factors were driven by a few patients, the disease factors were identified from 90% of randomly selected patients with the optimal K determined in the previous step. This procedure was repeated 100 times. Spearman's correlation coefficients between factor composition using all patients and using a patient subset were obtained, with the corresponding factors matched using the Hungarian matching algorithm.<br>It was also examined whether the framework could infer the factor composition in unseen patients. Disease factors (two positive and two negative) were obtained from the patients in the discovery dataset. Then, a linear regression model was constructed to predict the factor composition of the patients with depression in the validation dataset 1. Spearman's correlation coefficients between the predicted factor composition and the true factor composition (obtained using patients in the validation dataset 1) were calculated to measure the extendibility of the framework in unseen patients. The corresponding factors obtained from the discovery and validation datasets were matched using the Hungarian matching algorithm.<br>In the discovery ,factors obtained using all subjects show highly consistent with those using subgroups. What is more, our results demonstrated that the framework could significantly predict the factor composition of unseen patients.
Randomization	Randomization was not performed because participants. Participants were allocated into patient and control groups based on information on diagnosis.
Blinding	Blinding is not relevant to this study, because subjects used in this study were grouped based on whether they diagnosed as depression or not.

Reporting for specific materials, systems and methods

We require information from authors about some types of materials, experimental systems and methods used in many studies. Here, indicate whether each material, system or method listed is relevant to your study. If you are not sure if a list item applies to your research, read the appropriate section before selecting a response.

Materials & experimental systems

n/a	Involved in the study	
☒	☐	Antibodies
☒	☐	Eukaryotic cell lines
☒	☐	Palaeontology and archaeology
☒	☐	Animals and other organisms
☒	☐	Clinical data
☒	☐	Dual use research of concern

Methods

n/a	Involved in the study	
☒	☐	ChIP-seq
☒	☐	Flow cytometry
☐	☒	MRI-based neuroimaging

Magnetic resonance imaging

Experimental design

Design type	resting state
Design specifications	1 scan per participant
Behavioral performance measures	n/a

Acquisition

Imaging type(s)	structural
Field strength	3T
Sequence & imaging parameters	Discovery dataset: repetition time = 8,164 ms, voxel size = 1 × 1 × 1 mm3, inversion time = 900 ms, echo time = 3.18 ms, flip angle = 7 degrees, resolution matrix = 256 × 256, slices = 188, thickness = 1.0 mm.<br>Validation dataset 1: The replication dataset was acquired on a 3-Tesla GE Discovery MR750 scanner (General Electric, Fairfield Connecticut, USA). Structural T1-weighted images with 3D spoiled gradient echo scan sequence with the

following parameters: TR/TE = 5.92/1.956 ms, voxel size = 1 mm × 1 mm × 1 mm, slice thickness = 1 mm, no gap, flip angle = 12°, matrix size = 256 × 256, and 156 slices.
The validation dataset 2 was acquired on a 3.0-T Siemens Trio MRI scanner (Siemens Medical, Erlangen, Germany) using a magnetization-prepared rapid gradient echo (MPRAGE) sequence with the following parameters: repetition time = 1,900 ms, echo time = 2.52 ms, inversion time = 900 ms, flip angle = 90 degrees, resolution matrix = 256 × 256, slices = 176, thickness = 1.0 mm, and voxel size = 1 × 1 × 1 mm3.

Area of acquisition: Whole brain

Diffusion MRI: ☐ Used ☒ Not used

Preprocessing

Preprocessing software: The structural images were preprocessed using the Computational Anatomy Toolbox 12

Normalization: non-linear

Normalization template: MNI305

Noise and artifact removal: The Image Quality Rating (IQR) combing image noise contrast ratio, inhomogeneity contrast ratio and root mean square was recorded to assess data quality.

Volume censoring: Data was not volume censored.

Statistical modeling & inference

Model type and settings: Main results were obtained using individualized analysis method. The group-level differences were obtained using standard linear models were fun for each brain across subjects to determine t-statistic value for disease effects on brain morphology. Covariates were described in the Results and Methods for each sample. These continuous t-statistic values were used to evalute the relationship with individualized differences.

Effect(s) tested: N/a

Specify type of analysis: ☒ Whole brain ☐ ROI-based ☐ Both

Statistic type for inference (See Eklund et al. 2016): voxel-wise

Correction: FDR or permutating test (details were included in methods part)

Models & analysis

n/a	Involved in the study
☒	☐ Functional and/or effective connectivity
☒	☐ Graph analysis
☐	☒ Multivariate modeling or predictive analysis

Multivariate modeling and predictive analysis: we constructed a linear regression model to test whether group-level analysis differences (unthresholded T map) could be quantitatively inferred from the identified 4 disease factors. The group-level differences were assumed to represent an ‘average’ patient and the coefficients of the regression model were treated as its factor compositions.
